# Post-hysterectomy Vesicovaginal Fistula: A Case Report

**DOI:** 10.7759/cureus.77181

**Published:** 2025-01-09

**Authors:** Srishti Kalra, Anuja Bhalerao

**Affiliations:** 1 Department of Obstetrics and Gynaecology, NKP Salve Institute of Medical Sciences and Research Centre, Nagpur, IND

**Keywords:** o'connor's procedure, post-hysterectomy, total abdominal hysterectomy, urinary incontinence, vesicovaginal fistula

## Abstract

In many developing nations, vesicovaginal fistulas (VVF) continue to be a serious cause of concern. It leads to significant morbidity in urology for women. Patients of VVF experience social rejection as a result of constant dribbling, stink, and wetness, which degrades their quality of life. Hence, this report presents the case of a 45-year-old woman who approached the Outpatient Department of Obstetrics and Gynaecology with complaints of leakage of urine and urinary incontinence for three months with a surgical history of total abdominal hysterectomy. On per-speculum examination, leakage of urine was observed, and on per-vaginal speculum, a rent of 1×2 cm was palpable on the anterior wall of the vagina. For diagnostic assessment, cystoscopy and computed tomography urography confirmed the diagnosis of post-hysterectomy VVF. The surgical intervention involved cystotomy for the supratrigonal fistula, which was 1.5×1.5 cm in size, and the repair was performed by O'Connor's procedure. In conclusion, appropriate preoperative diagnosis, investigations, and principles of surgical intervention lead to improved surgical outcomes.

## Introduction

The Global Burden of Disease research conducted by the World Health Organization found that unfavorable maternity-related factors accounted for 14.5 quality-adjusted years per woman and disability-adjusted life years lost by women between the ages of 15 and 44 were due to reproductive-related health causes which accounted for 21.9% years. Twenty-two percent of all morbid maternal diseases were related to obstructed labor, which is the leading cause of maternal death. Moreover, maternal morbidity is correlated with obstetric fistulas [1].

Vesicovaginal fistula (VVF) is considered one of the most discomforting consequences of gynecologic and obstetric procedures leading to an erratic opening between the vagina and the bladder that causes relentless and persistent urine incontinence. The social resentment associated with VVF causes women to suffer silently, making it impossible to estimate the precise incidence. The incidence and etiology of VVF vary across the globe [1]. The surgical procedures of the pelvis more often serve as the causative factor of VVF with an incidence range between 0.3% and 2% in developed countries [2,3]. Treatment for advanced pelvic malignancies including cervical, rectal, and bladder cancers and those caused by radiation are less frequent causes of VVF [1,2].

Women with VVF experience social stigma which degrades their quality of life and substantial development. These women lose their potential for societal advancement and success during their most productive years [3]. Despite being a frequently mentioned condition, there are not enough well-conducted or well-established management guidelines described in the literature. Hence, the present case report aims to highlight the prevention, early diagnosis, and proper management of VVF.

## Case presentation

Patient information

A 45-year-old woman reported to the Outpatient Department of Obstetrics and Gynaecology with primary complaints of leakage of urine and urinary incontinence for three months. The patient described a surgical history of total abdominal hysterectomy for abnormal uterine bleeding at a local hospital, eight days before the onset of symptoms. The obstetric history of the patient reported three parity and two abortions (G5P3L3A2) with all three deliveries through normal vaginal deliveries following a standard duration of hospital stay following the normal vaginal deliveries (3-4 days).

Clinical examination

The patient was vitally stable. On per-speculum examination, leakage of urine was observed; however, the vaginal vault was healthy. On per-vaginal speculum, a rent of 1×2 cm on the anterior vaginal wall was palpable.

Diagnostic assessment

**Figure 1:**
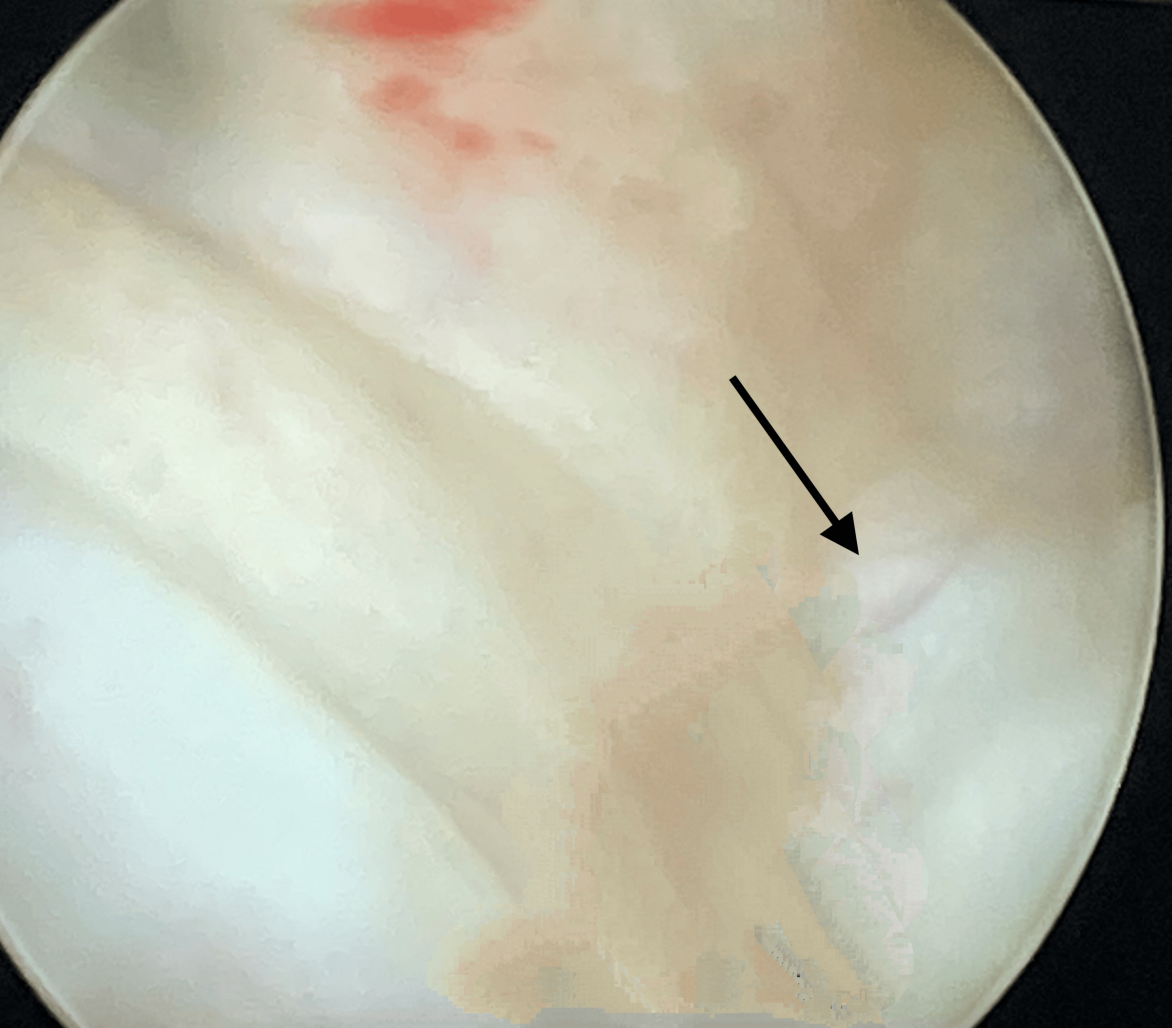
Cystoscopy using an F-20 sheath Black arrow: 1×1.5 cm supratrigonal vesicovaginal fistula

A cystoscopy was performed using an F-20 sheath for diagnostic assessment. The findings demonstrated a 1×1.5 cm supratrigonal VVF proximal to the inter-ureter base, as illustrated in Figure 1. Hence, the diagnosis of post-hysterectomy VVF was confirmed.

Therapeutic intervention

Due to the supratrigonal bladder position of the fistula, repair through the abdominal route was planned. Introduction of per-urethral Foley's catheter was performed, and 30 ml of normal saline in its bulb was instilled. To delineate the bladder, the retropubic space was dissected, and a cystotomy of 3 cm was performed. Ureteric stents were placed and the diverticulum was located.

**Figure 2:**
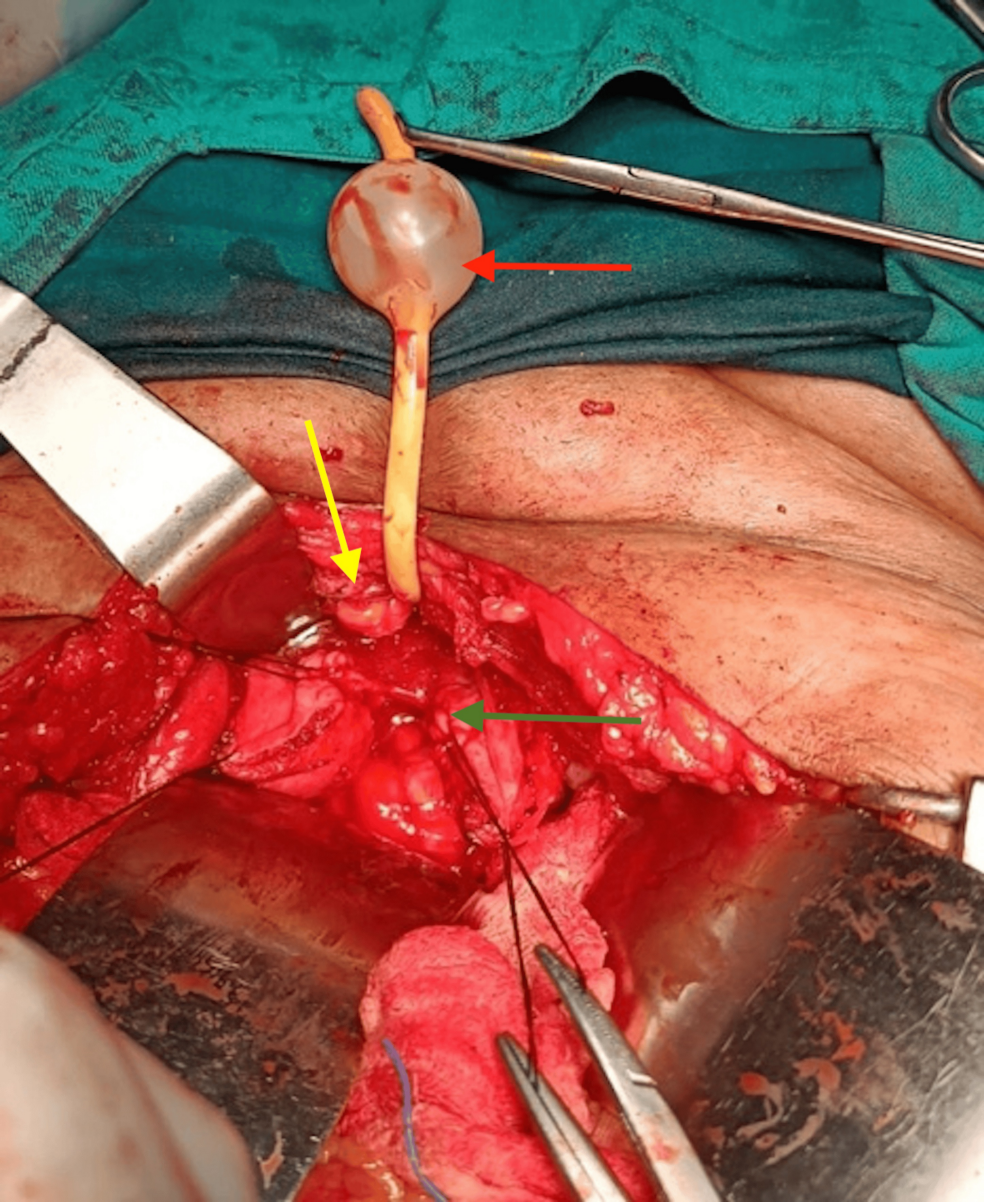
Intraoperative illustration during O'Connor's procedure Yellow arrow: anterior wall of the bladder; green arrow: posterior wall of the bladder; red arrow: Foley's bulb

In the midline, the fistula was 1.5×1.5 cm in size, so O'Connor's procedure [4] was decided for repair. The procedure involved appropriate mobilization on the anterior-superior and posterior planes, followed by which the bladder was bisected until the end of the fistula with a vertical incision. From the vagina, the bladder walls surrounding the fistula were released. The anterior wall of the bladder had been opened following the separation of the fistula from the vagina, and the posterior wall of the bladder was repaired as shown in Figure 2. The repair was executed in two layers using delayed absorbable sutures and an interposed omental flap between the vagina and the bladder. Additionally, the suprapubic catheter was inserted, and for an arbitrary period of three weeks, continuous drainage of the bladder was ensured. After two weeks, all the symptoms of the patient had resolved, and she was continent and healthy.

## Discussion

VVF is much rarer in developed nations, where it originates primarily as a consequence of surgical procedures of the pelvis like hysterectomy or following radiotherapy for malignancy [5]. In their systematic review, Hillary et al. found that only 4.8% of the cases of VVF in underdeveloped nations had surgical origins, whereas 83.2% of incidents in developed nations had its origin as a consequence of surgical procedure pelvis ranging from abdominal hysterectomy or benign and malignant urological, colorectal, and gynecological procedures [6]. Of all fistulas, 75% are the result of VVFs after abdominal hysterectomy. Most often, an inappropriate suture or clamp inserted into the bladder wall after surgery or an unnoticed injury to the bladder serves as the precipitating factor for VVF. Injury to the ureter can be prevented by the use of intracapsular techniques, with the ligature and sectioning of cardinal ligaments, uterosacral and vesicouterine, as close as possible to the uterus. The sutures should be taken under the proper visualization of the tip of the clamp. The ureterovesical fold should be opened properly, and the bladder should be pushed down. Hysterectomies are considered to contribute to 0.5-2% of VVFs [7]. Similarly, in the present case, the patient had a surgical history of total abdominal hysterectomy followed by VVF.

In the present report, a diagnosis was made based on cystoscopy findings. Literature assessing the reliability of cystoscopy revealed a 92-93% sensitivity rate for the diagnosis of VVF [8]. In a study conducted by Sohail and Siddiqui, urine incontinence in 15 individuals was diagnosed or suspected to be caused by a VVF [9]. The sensitivity of cystoscopy was observed to be 93% [10].

VVF management is still considered a challenge due to the lack of appropriate guidelines. Due to the supratrigonal position of the fistula, repair using O'Connor's procedure via the abdominal route was planned in our case. A study by Singh et al. on the repair of VVF by the trans-abdominal route in a north Indian tertiary hospital reported a success rate of 87.5% [11]. As the traditional O'Connor approach provides excellent omental interposition and mobilization of tissues, it is considered to be the most accepted method of supratrigonal VVF treatment [12]. However, the extravesical technique was also reported as a safe, effective, and minimally invasive technique with excellent cure rates for VVF [13]. Similarly, robotic-assisted laparoscopic VVF repair was reported as an effective and safe treatment option [14].

The repair in the present case was done in two layers using delayed absorbable sutures and an interposed omental flap between the vagina and the bladder. In trans-abdominal repair, for placing an interposition flap, the omentum remains the first choice. The principles of omental flap mobilization were described in 1967 by Turner-Warwick et al. according to the right gastroepiploic artery coming from the stomach's greater curvature. Omentum has favorable vascularity and prevents the two suture lines from friction when it is positioned between the bladder and the vagina [15]. Omentum flaps are therefore utilized in cases of complicated, malignant, and complex fistulas. Nonetheless, since the surrounding tissues are healthy and well-vascularized, interposition flaps could be safely skipped in cases of a simple fistula [16]. According to Evans et al., the interposition flap in a trans-abdominal approach had a 100% success rate for benign and malignant fistulas, while the failure rate without the flap was 63% for benign fistulas and 67% for malignant fistulas [17].

A thorough preoperative evaluation, extensive exposure of the fistula and surrounding tissues with excision of the fibrotic tissue, tension-free closure, avoiding undue tension on the vaginal mucosa, and preservation of an uninfected and dry suture are all factors that boost the repair rate of a VVF [17]. In comparison to vaginal closure to accomplish a favorable outcome, bladder closure appears to be more relevant which was achieved in the present case.

## Conclusions

The present case highlighted VVF which is the most distressing complication of gynecological and obstetric procedures. However, they are preventable and treatable, and the crucial factors include early detection, avoidance of prolonged labor, and careful performance of gynecological surgeries. This can be implemented with improvement in obstetric care in developing countries. Prevention-related measures must include enhanced and accessible medical services along with universal education related to VVF complications involving a thorough symptom and care-guide to the patient. However, traditional surgical management remains the preferable option. Hence, future studies focusing on the development of new treatment approaches are essential for improving the quality of life among women.
